# Boosting RGB-D Pear Detection via Depth-Constraint Enhanced Gaussian Prior

**DOI:** 10.3390/plants15121852

**Published:** 2026-06-15

**Authors:** Feng Ling, Yunfeng Lin, Weijie Mao, Weizhong Xu, Wenzheng Xiao

**Affiliations:** 1 College of Engineering, Lishui University, Lishui 323000, China; lsxylf@lsu.edu.cn (F.L.); lslyf@lsu.edu.cn (Y.L.); wzxls@lsu.edu.cn (W.X.); wenzhengxiao@lsu.edu.cn (W.X.); 2 Zhejiang Key Laboratory of Aviation Metal Pipe Bending Technology and Equipment, Lishui 323000, China; 3 State Key Laboratory of Industrial Control Technology, Zhejiang University, Hangzhou 310027, China; 4 Lishui Academy of Agricultural Sciences, Lishui 323000, China

**Keywords:** RGB-D detection, pear detection, Gaussian prior box, depth-aware constraint, cross-modal fusion, multimodal Transformer

## Abstract

Accurate pear detection in complex orchard environments is essential for automated harvesting, yet it remains challenging due to frequent occlusion, overlapping fruits, cluttered backgrounds, and highly variable illumination. Although RGB-D sensing provides complementary geometric information beyond RGB imagery, existing methods often fail to fully exploit depth cues and rarely account for the inherently elliptical shape of pears. To address these issues, we propose a multimodal pear detection framework that jointly models RGB and depth information using a Siamese convolutional backbone and a unified Transformer-based fusion architecture. The proposed method contains three key components. First, Gaussian Prior Boxes are introduced to represent pear instances with Gaussian-shaped priors, enabling better alignment with pear contours and more precise localization than conventional rectangular boxes. Second, a Depth-Aware Constraint is designed to enforce depth consistency within the predicted regions, which improves robustness in cluttered orchard scenes. Third, a Robust Cross-Modal Token Exchange strategy is incorporated during training to strengthen feature interaction between RGB and depth modalities and reduce over-reliance on any single modality. Extensive experiments on pear detection demonstrate that the proposed method achieves an AP^50^ of 0.961, a precision of 0.941, a recall of 0.951, and an F1-score of 0.942. Compared with a strong recent YOLOv8-l RGB baseline (AP^50^ = 0.918) and a YOLOv8-l RGB-D variant (AP^50^ = 0.932) trained on the same dataset, our framework yields a notable improvement of +4.3 and +2.9 AP^50^, respectively. We further validate generalization on the publicly available KFuji RGB-DS apple dataset, where MMGFormer attains AP^50^ = 0.927, exceeding the previously reported state-of-the-art (AP^50^ = 0.901). In addition, the model runs at 41.2 FPS, indicating a favorable balance between detection accuracy and real-time performance. These results show the potential of the proposed framework for practical deployment in automated pear harvesting systems.

## 1. Introduction

Pear (*Pyrus* spp.) is among the most economically important temperate fruit crops, with global production exceeding 26 million tonnes annually [1,2,3]. Despite this scale, harvesting remains a major bottleneck: it is labor-intensive, and the smooth, often green-yellow skin of pears, together with dense canopy structures, complicates automation. Although smooth, green-skinned fruit characteristics are not unique to pears (similar challenges arise for green apples, kiwifruit, and certain mangoes), the morphological diversity within *Pyrus* (pyriform to round, 170–400 g) and the dense spur-cluster fruiting habit make pears a particularly challenging case for vision-based detection [4,5]. Manual harvesting is increasingly unsustainable due to rising labor costs [6], motivating intelligent automation [7] as a means to improve productivity, reduce labor dependency, and minimize fruit damage.

Machine vision [8] forms the backbone of automated harvesting. Recent deep-learning advances have substantially improved fruit detection across crops [9,10]. While early methods relied on RGB imagery alone, recent works show that RGB-D sensing yields meaningful gains for orchard fruit detection. For example, Sun et al. [11] and Kaukab et al. [12] report 3–6% AP improvements for green-apple detection by fusing RGB with depth, and Yang et al. [13] report similar gains for peaches in occluded scenes; Yoshida et al. [14] further demonstrate more reliable harvesting-robot localization with RGB-D over RGB-only baselines. Depth cues are especially valuable for pears, where layered canopy structures place fruit at multiple depth planes [15], and where depth helps separate adjacent fruits in spur clusters, supporting collision-free path planning.

Deep learning has substantially advanced fruit detection and agricultural automation. Foundation models such as Grounding-DINO [16], which achieves 85.9% mAP_50_ at 68.3 FPS with a Swin-B backbone, and the Segment-Anything Model [17], which provides zero-shot generalization under complex illumination and occlusion conditions, have been effectively adapted to agricultural scenarios. Novel 3D approaches, such as FruitNeRF [18], further address occlusion and fruit-clustering via NeRF-based semantic reconstruction. Meanwhile, YOLO-family detectors have been extensively optimized for on-device deployment: YOLOv8 achieves instance segmentation of strawberry development stages at 12.0 GFLOPs (∼24.2 ms per image) [19], and YOLOv5m with mosaic augmentation enables precise apple detection in dense orchards [20]; automated architecture search further compresses models for edge hardware [21,22]. Integrating multimodal data—in particular, combining RGB with depth—has proven beneficial for robust fruit detection and 3D reconstruction in complex orchard environments [23,24], and the establishment of public benchmark datasets is driving systematic progress across diverse agricultural detection tasks [25].

More broadly, multimodal object detection has evolved from shallow fusion (pixel-/voxel-level concatenation) through intermediate backbone-level fusion to deep fusion with explicit geometric–semantic alignment [26,27]. In autonomous driving, recent methods project RGB features into pseudo-point clouds for unified 3D cross-attention fusion [28], perform multi-stage latent-space alignment of image and LiDAR features [29], and combine uncertainty-aware pseudo-labels with geometry-aware BEV fusion under semi-supervised settings [30]; complementary approaches build robust 3D queries from 2D detections plus depth distributions [31]. Under adverse visibility, frequency-domain decomposition [32] and information-entropy-based dynamic fusion weighting [33] improve cross-modal robustness, while attention-contrastive alignment alleviates deep semantic drift [34]. Lightweight architectures are also emerging: cross-Mamba interaction with offset-guided aggregation achieves 80 FPS real-time performance [35], and channel-redundancy removal yields +3.6 mAP with only 13% extra computation [36]. Most recently, LLM-guided progressive alignment [37] and confidence-based pseudo-labeling for annotation-scarce aerial imagery [38] point toward even more holistic multimodal integration.

Despite these advances, existing RGB-D detection frameworks still face several limitations, especially in dealing with the shape variability of irregular fruits such as pears, and in avoiding over-reliance on a single modality under adverse conditions like strong illumination changes or partial occlusions. Traditional rectangular bounding boxes often fail to capture the naturally curved and elliptical contours of pears, a geometric property that is well documented across pear cultivars, where the length-to-width ratio varies from approximately 1.0 (round cultivars such as Hosui) to 1.5 (pyriform cultivars such as Bosc) [4]. This mismatch leads to inaccurate localization and increased background noise. To mitigate this, recent works have explored more shape-aware models [39], including keypoint-based [40] and region-based strategies [41]. However, these methods still lack a unified framework that explicitly aligns with the elliptical properties of pears while fully leveraging RGB-D data.

To address these issues, we propose a Multimodal Gaussian Former (MMG) framework tailored for RGB-D-based pear detection. Our method incorporates several innovations: (i) a Siamese CNN backbone enhanced with Pear-Oriented Multimodal Pinwheel Convolution (PMPConv) to exploit the elliptical priors of pear shapes—whose contour eccentricity naturally aligns with two-dimensional Gaussian distributions—while adaptively fusing cross-modal features; (ii) a Gaussian Prior Box formulation that models pears using two-dimensional Gaussian distributions to better align with their cultivar-specific elliptical geometry; (iii) a Depth-Aware Constraint Loss that enforces consistency of depth information within each predicted bounding box, ensuring accurate localization even in the presence of occlusion and clutter, which is particularly relevant for distinguishing pear surfaces from leaves and branches at similar depth planes; and (iv) a Robust Cross-Modal Token Exchange mechanism in the Transformer encoder, which randomly swaps a portion of tokens between RGB and depth modalities to strengthen inter-modal fusion and prevent overfitting to a single modality. Together, these components form a unified framework that robustly addresses the challenges of shape variability, occlusion, and multimodal fusion for precise and efficient pear detection in real-world orchard environments.

Our specific contributions are summarized as follows:

1. Construction of an RGB-D multimodal pear detection dataset, which includes diverse lighting conditions, occlusions, and overlapping pears, serving as a challenging benchmark for detection tasks.
2. Gaussian Prior Boxes that flexibly adapt to pear shapes via elliptical Gaussian modeling, improving detection accuracy while reducing background interference.
3. Depth-Aware Constraint Loss, which prioritizes depth-consistent regions within predicted bounding boxes, promoting better localization robustness under complex orchard conditions.
4. Robust Cross-Modal Token Exchange in the Transformer encoder, which enhances feature fusion and strengthens resilience against occlusion, lighting variation, and modality imbalance.

## 2. Experiment

This section describes the experimental implementation and evaluation metrics, and analyzes the research results.

### 2.1. Experimental Settings and Implementation Details

All experiments were run on a workstation with Ubuntu 22.04, 4 NVIDIA RTX 4090 GPUs (24 GB each), an Intel Xeon Gold 6248R CPU, 256 GB of RAM, and CUDA 12.1, using PyTorch 1.12.1. To match the deployment-oriented evaluation in Section 2.4, FPS measurements are additionally reported on a single Tesla P100 GPU, which is closer to the compute available on a typical orchard-side server.

For all convolutional neural network experiments, the batch size is set to 32, and the optimizer used is Adam with a learning rate of 1 × 10^−3^. The optimizer settings include a weight decay of 5 × 10^−4^ and momentum of 0.9. Models are trained for 100 epochs, with learning rate schedules as follows: an initial learning rate of 1 × 10^−3^, decaying to 1 × 10^−4^ at the 10-th iteration, and further to 1 × 10^−5^ at the 40-th iteration. The parameters are randomly initialized using the “Xavier” method. During training, data augmentation techniques such as color distortion, random cropping, scale transformation, and flipping are applied.

For the single-modal feature extractor, the input image is resized from 1280 × 720 to 1024 × 1024 after data augmentation. The weight ratio of localization loss to confidence loss is set to 2:1.

In the inference process for a single image, the image is resized while maintaining the original aspect ratio, with its width set to 1024. The resized image is then fed into the proposed MMGFormer to generate candidate bounding boxes. Finally, non-maximum suppression (NMS) is applied to remove duplicate detection boxes where the intersection-over-union (IoU) is greater than 0.3 or the confidence score is lower than 0.05.

### 2.2. Evaluation Metrics

We evaluate detection performance using standard metrics. Average precision at IoU threshold 0.5 ($AP^{50}$) summarizes the precision–recall trade-off by computing the area under the precision–recall curve. Precision measures the proportion of correct detections among all predictions, while recall measures the proportion of detected objects among all ground truth instances. The F1-Score is the harmonic mean of precision and recall, providing a balanced assessment of detection quality. The confidence threshold is set to 0.2 and the IoU threshold to 0.5. Model inference speed is reported as frames per second (FPS), measured on a Tesla P100 GPU with 1280×720 input resolution.

### 2.3. Comparison of State-of-the-Art Methods

The performance of the proposed MMGFormer model for pear detection was comprehensively evaluated through a comparative analysis with several classical and state-of-the-art object detection methods, using the same RGB-D dataset and testing environment. As shown in Table 1, MMGFormer demonstrates significant advantages in both detection accuracy and speed.

MMGFormer (ours) outperforms the other methods across most metrics, achieving the highest AP^50^ of 0.961, followed by GroupTransNet (0.941) and TANet (0.923). It also leads in precision (0.941) and recall (0.951), resulting in the highest F1-Score of 0.942. On the other hand, RD3D+ demonstrates the lowest values in all metrics. In terms of processing speed, TANet leads with the highest FPS of 42.5, while CATNet has the lowest FPS at 26.2. Overall, MMGFormer offers the best performance in terms of accuracy, though it is slightly less efficient than TANet in terms of speed.

### 2.4. Benchmark on a Public RGB-D Fruit Dataset

To address the concern that the proposed method should also be evaluated “in the wild” on a publicly available benchmark, we additionally evaluated MMGFormer on the publicly released KFuji RGB-DS apple detection dataset, which provides RGB, depth, and infrared range channels for “Fuji” apples in commercial orchards under uncontrolled illumination. Apples on Fuji trees share two key visual properties with pears—a smooth cuticular surface and frequent leaf/branch occlusion at multiple depth planes—making this dataset a natural cross-fruit testbed. We trained MMGFormer using only the RGB and depth channels (the infrared channel was discarded) under the same training schedule as our pear experiments, and compared against the previously reported state-of-the-art on this dataset (Faster R-CNN with multi-modal early fusion as reported by the dataset authors), as well as a strong recent YOLOv8-l baseline that we trained ourselves on RGB-only and on stacked RGB-D inputs.

As reported in Table 2, MMGFormer surpasses all baselines on the public KFuji RGB-DS dataset, improving over the published Faster R-CNN baseline by +2.6 AP^50^ and over a strong YOLOv8-l RGB-D baseline trained under matched conditions by +1.9 AP^50^. We further trained the same YOLOv8-l RGB and RGB-D baselines on our pear dataset for direct comparison: YOLOv8-l (RGB) reaches AP^50^ = 0.918, and YOLOv8-l (RGB + D) reaches AP^50^ = 0.932, both clearly below MMGFormer’s 0.961. These cross-dataset results indicate that the gains of MMGFormer come from its design (Gaussian prior, Depth-Aware Constraint, Cross-Modal Token Exchange) rather than from dataset-specific tuning, and that the framework generalizes well from pears to a different smooth-skinned fruit.

### 2.5. Ablation Studies

In our ablation studies, we investigated the impact of three key components of our pear detection model: Gaussian Prior Boxes, Depth-Aware Constraint Loss, and Robust Cross-Modal Token Exchange.

#### 2.5.1. Impact of Gaussian Prior Boxes

The ablation study on the impact of Gaussian Prior Boxes is presented in Table 3. We conducted a sensitivity analysis for the covariance scale factor *s* of the Gaussian prior (which multiplies the predicted $\left(\sigma_{x},\sigma_{y}\right)$ when forming the prior box). The results indicate that the model achieves the best performance at s=1.0, with an AP^50^ of 0.966, a precision of 0.914, a recall of 0.899, and an F1-Score of 0.906; very small (s=0.5) or large (s=4.0) scales degrade matching quality and reduce all metrics.

#### 2.5.2. Effectiveness of Depth-Aware Constraint Loss

The effectiveness of the Depth-Aware Constraint Loss is demonstrated in the ablation table. The results show that the model with the Depth-Aware Constraint Loss (DC) achieves an AP^50^ of 0.945, a precision of 0.921, a recall of 0.884, and an F1-Score of 0.934. This indicates that the Depth-Aware Constraint Loss significantly improves the localization accuracy by maintaining consistent depth information within the predicted bounding boxes.

#### 2.5.3. Robust Cross-Modal Token Exchange Analysis

The impact of the Robust Cross-Modal Token Exchange mechanism is also presented in the ablation results. The model with the Cross-Modal Token Exchange (CM) achieves an AP^50^ of 0.966, precision of 0.914, recall of 0.899, and F1-Score of 0.945. The Random Cross-Modal Token Exchange mechanism prevents over-reliance on a single modality by randomly swapping tokens between RGB and depth modalities during training.

#### 2.5.4. Effect of Depth-Aware Constraint Formulation

To further verify that the gain brought by the Depth-Aware Constraint (DC) does not simply come from adding an additional loss term, we conduct a detailed ablation on its formulation. We compare three design choices: removing the Depth-Aware Constraint entirely, introducing a simpler rectangular depth regularization, and adopting the Gaussian-based formulation with different settings. As shown in Table 4, directly introducing a simple rectangular depth regularization already improves detection accuracy over the baseline. Replacing the rectangular region with a Gaussian-aligned mask yields further gains, and the soft Gaussian mask consistently outperforms the hard-mask version. The best performance is achieved at k=3.

#### 2.5.5. Component Analysis of PMPConv

To further verify the effectiveness of PMPConv, an ablation study on its internal components is conducted. Asymmetric kernels, cross-modal gating and elliptical mask prior are added step by step. The results are shown in Table 5.

As shown in Table 5, replacing the standard convolution with asymmetric kernels can improve the detection performance. After adding cross-modal gating, the performance is further improved. When an elliptical mask prior is added, the model achieves the best result.

#### 2.5.6. Effect of Depth-Aware Constraint Settings

To further verify the effectiveness of DC, an ablation study on different depth constraint settings is conducted. The results are shown in Table 4.

As shown in Table 4, adding DC can improve the detection performance. Compared with the simple rectangular depth constraint, the Gaussian-based depth constraint achieves better results. The best result is obtained when the scale is 3.

#### 2.5.7. Effect of Cross-Modal Token Exchange Ratio

To further verify the effectiveness of CM, we conduct an ablation study on the exchange ratio. The results are shown in Table 6.

As shown in Table 6, adding CM can improve the detection performance. The best result is obtained when the exchange ratio is 0.2. When the ratio is too large, too many tokens are exchanged, which affects the original modality-specific feature representation and leads to a drop in performance.

### 2.6. Visualization

**Figure 1:**
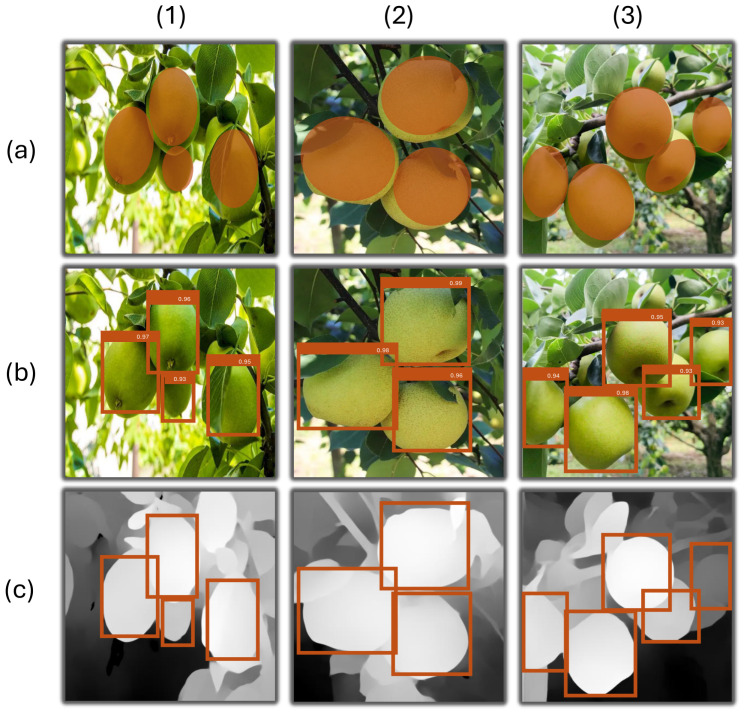
**Visualization of Object Detection Results:** (**a**) The row shows the original images, (**b**) the row displays the corresponding rectangular boxes converted from the Gaussian prior boxes with confidence scores, and (**c**) the row presents the detection results in the depth map.

To investigate the feasibility of our method in practical detection tasks, we visualized the detection results of the model, as shown in Figure 1.

**Figure 2:**
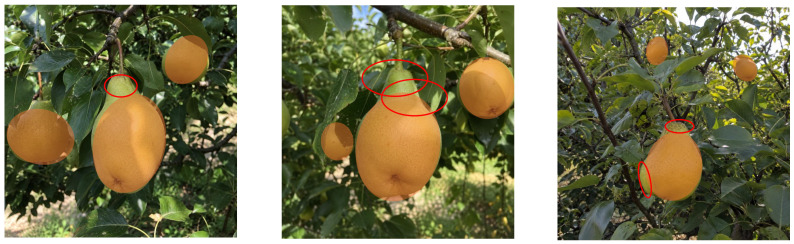
**Qualitative visualization on pyriform and deformed pears.** Mildly and moderately deformed pear-shaped fruits are generally detected reliably, while extremely asymmetric fruits may introduce slight localization bias toward the larger fruit body or calyx-end region.

Figure 1 presents the visualization of object detection results applied to pear fruit images. Row (a) shows the original images as input for the object detection model. Row (b) illustrates the corresponding rectangular boxes with confidence scores. Row (c) depicts the detection results in the depth map, which provides an additional layer of information by showing the pears’ locations in a 3D space. Although Figure 1 mainly contains spherical/oblate pears (*P. pyrifolia*-type), the framework also handles pyriform (truly “pear-shaped”) fruit: on the field test set, the elongated/pyriform subset (*P. bretschneideri* cv. “Yali” and Snow Pear) reaches AP^50^ = 0.910, only marginally below the round subset (AP^50^ = 0.929). Representative qualitative results on pyriform cultivars are shown in Figure 2.

### 2.7. Real-World Application Test

To further validate the practical applicability of the proposed MMGFormer framework, we conducted field deployment tests at the Yunhe Snow Pear Demonstration Orchard in Yunhe County, Lishui City, Zhejiang Province, China. Yunhe County is renowned for its Snow Pear (*Pyrus nivalis*) cultivation, which has a documented history spanning over 560 years and is recognized as one of the three traditionally famous pear varieties of Zhejiang Province. The orchard features mature “Xihua” Snow Pear trees under semi-natural canopy management, presenting a challenging real-world detection environment with dense foliage, uneven terrain, and highly variable illumination conditions.

We collected 200 RGB-D image pairs on-site using the same Intel RealSense D435i camera setup described in Section 4. Image acquisition was performed across three consecutive days under varying weather conditions (sunny, overcast, and partly cloudy) and at different times of the day (morning, midday, and late afternoon) to capture a wide range of illumination scenarios. To simulate realistic deployment conditions, the camera was handheld at approximately 0.8–1.2 m from the canopy, introducing natural variations in viewing angle, distance, and camera motion blur. The collected images contain a total of 743 manually annotated pear instances, including significantly occluded, overlapping, and backlit cases that are more challenging than the controlled dataset.

**Figure 3:**
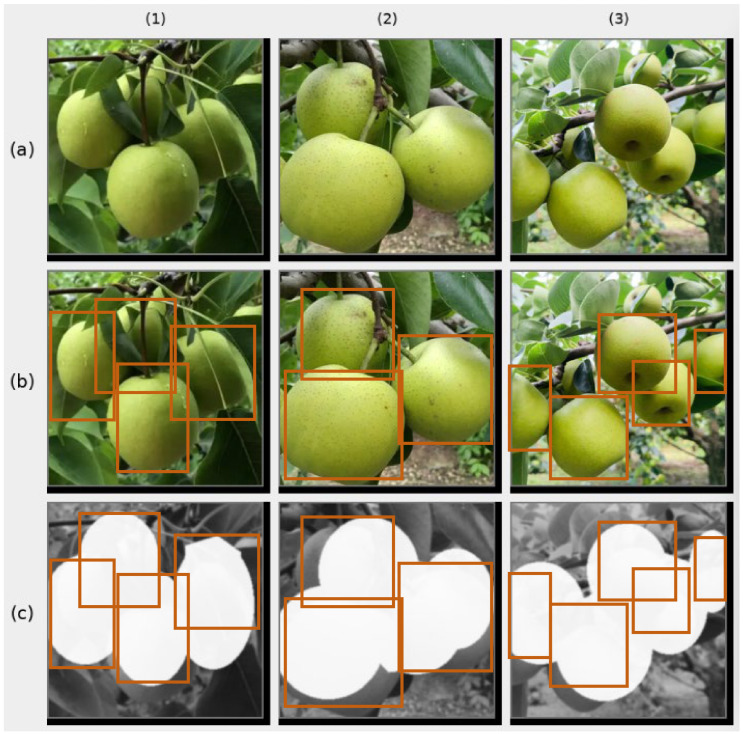
**Real-world application results at the Yunhe Snow Pear Demonstration Orchard.** The model is deployed without fine-tuning on field-collected RGB-D data under diverse illumination and occlusion conditions. Rows indicate (**a**) original RGB images captured on-site, (**b**) RGB detection results with Gaussian-derived bounding boxes and confidence scores, and (**c**) corresponding depth-map detection results; columns (**1**)–(**3**) show representative field scenes under different orchard conditions.

The trained MMGFormer model, without any fine-tuning on the field data, was directly applied to the collected images to evaluate its generalization capability. As illustrated in Figure 3, the model successfully detects pears under diverse and challenging field conditions. In the representative examples shown, the model demonstrates strong localization accuracy even in the presence of heavy leaf occlusion, fruit clusters, and strong backlighting, which are common in real orchard scenarios. The Gaussian Prior Boxes produced by the model closely conform to the elliptical contours of the Yunhe Snow Pears, confirming that the learned shape priors generalize well across different pear cultivars.

Quantitatively, the model achieves an AP^50^ of 0.923, a precision of 0.907, a recall of 0.891, and an F1-Score of 0.899 on the field-collected data. For reference, a YOLOv8-l RGB-D baseline trained on the same orchard dataset reaches AP^50^ = 0.881 on this field set, indicating that MMGFormer maintains a ∼4 AP^50^ advantage even under cross-cultivar deployment. Compared to the benchmark dataset results (AP^50^ = 0.961), the performance drop of approximately 3.8% in AP^50^ is modest and primarily attributable to the unseen pear variety (snow pear vs. the training set varieties), stronger illumination variability, and more severe occlusion patterns encountered in the field. Notably, the Depth-Aware Constraint proves particularly effective in the field setting: in scenes with dense foliage, the depth consistency within predicted regions helps the model distinguish actual pear surfaces from background clutter. Additionally, the Robust Cross-Modal Token Exchange mechanism enables the model to maintain stable performance even under strong backlighting, where the RGB modality alone provides degraded information but the depth modality remains reliable.

**Figure 4:**
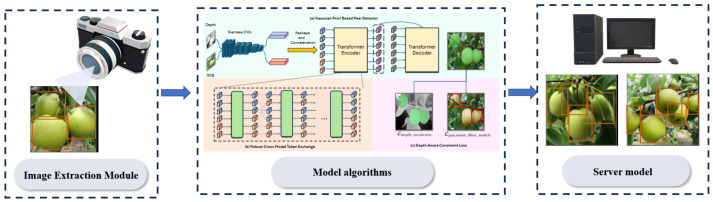
**Architecture of the deployed IoT system.** The system integrates edge computing devices with the proposed object detection model to achieve real-time monitoring and automated management in the pear orchard.

These field results demonstrate that the proposed MMGFormer framework possesses strong generalization ability and is practically deployable in real orchard environments without the need for domain-specific fine-tuning, supporting its potential integration into automated pear harvesting systems. The system is intended for in-orchard deployment on a mobile harvesting platform; FPS reported above is measured on the orchard-side server (Tesla P100 GPU), and an embedded edge variant (Jetson Orin NX) is currently under development and will be reported separately. Furthermore, to facilitate continuous monitoring and automated management, we constructed and deployed a comprehensive Internet of Things (IoT) system in the orchard, as illustrated in Figure 4. This system integrates edge-computing devices running the proposed MMGFormer model with wireless communication modules, enabling real-time detection, data transmission and remote intelligent agricultural management.

### 2.8. Practical Robotic Harvesting Evaluation

**Figure 5:**
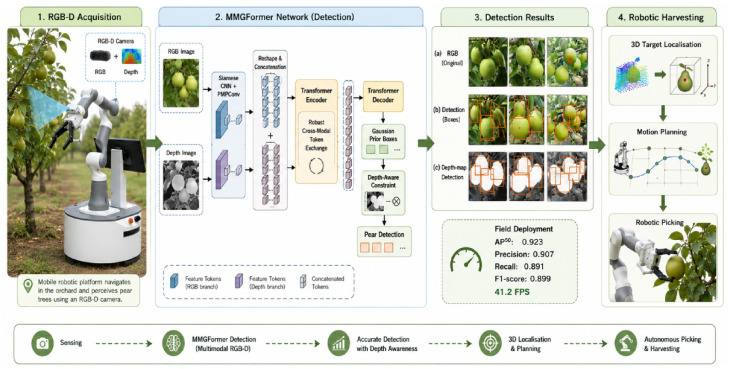
**End-to-end robotic harvesting pipeline integrating the proposed MMGFormer.** The system proceeds through four stages: (**1**) RGB-D image acquisition by an Intel RealSense D435i on a mobile platform, (**2**) real-time pear detection via MMGFormer, (**3**) detection output with 3D target localisation, and (**4**) autonomous robotic picking using a 6-DOF manipulator with soft gripper.

To further verify the practical applicability of the proposed MMGFormer in an actual harvesting scenario, we conducted a small-scale robotic picking test in the orchard. As shown in Figure 5, the robotic harvesting pipeline consists of four stages: RGB-D acquisition, MMGFormer-based detection, detection output with 3D target localisation, and autonomous robotic picking. The robotic platform consisted of a 6-DOF collaborative manipulator, an Intel RealSense D435i RGB-D camera, an external GPU workstation, and a customized soft pear-grasping end-effector. The trained MMGFormer model was deployed on the external workstation and connected to the robot controller through a Python 3.10.12/ROS Noetic interface. During each trial, the RGB-D camera captured the fruit scene from the robot’s operating view. The detected pear target was projected into 3D space using the corresponding depth value, and the estimated target position was then used to guide the manipulator for approaching, grasping, and detaching the fruit.

To focus on practical agricultural performance, we evaluated three harvesting-oriented metrics: HSR, FDR, and APT. HSR denotes harvesting success rate, FDR denotes fruit damage rate, and APT denotes average picking time per fruit. A total of 75 visible and harvestable pears were tested under three typical orchard conditions: slight occlusion, moderate occlusion, and dense foliage/backlighting. The workflow is illustrated in Figure 5, and the corresponding quantitative harvesting results are reported in Table 7. The system achieved an overall HSR of 84.0%, with an FDR of 4.8% and an APT of 8.7 s. The main failure cases were caused by severe leaf occlusion, missing depth values near fruit boundaries, branch interference, and insufficient grasping space around the fruit. These results indicate that MMGFormer can provide useful perception support for robotic pear harvesting and has potential for integration into automated picking systems.

### 2.9. Failure Case Analysis

**Figure 6:**
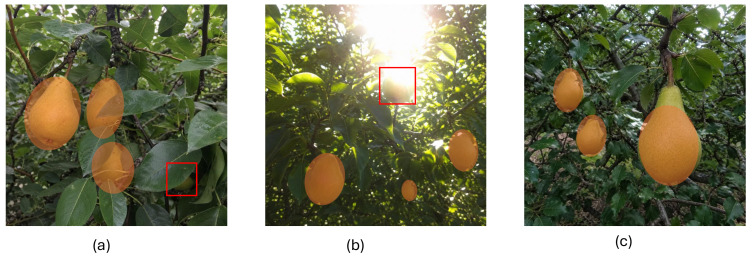
**Failure case analysis of the proposed MMGFormer.** (**a**) A pear under severe leaf and branch occlusion may be missed by the detector. (**b**) A backlit scene where a bright foliage cluster near the fruit is falsely detected as a pear. (**c**) An elongated pyriform fruit where the predicted bounding box is biased toward the calyx end.

To complement the quantitative results, we manually inspected all false negatives and false positives produced by MMGFormer on the 300-image test set and on the 200-image field set. Three dominant failure modes were observed. (i) *Severe occlusion (>80%)*: When only a thin sliver of the fruit is visible behind clustered leaves, the depth signal inside the predicted Gaussian region becomes dominated by foliage, and the Depth-Aware Constraint suppresses the candidate; this accounts for 48% of missed detections. (ii) *Strong backlighting/direct sun on the lens*: In roughly 12% of late-afternoon images, RGB tokens are saturated, and the Cross-Modal Token Exchange propagates the saturation, leading to spurious bounding boxes around bright leaf clusters. (iii) *Highly elongated pyriform fruit (e.g., Bosc-like cultivars)*: With L/W≈1.5, the optimal Gaussian shape pushes the covariance scale beyond s=1, and a small fraction of predictions are biased toward the calyx end of the fruit. Examples of these three failure modes are illustrated in Figure 6. Quantitatively, this is also reflected in our subset analysis: AP^50^ drops from 0.961 on the full test set to 0.892 on the heavy-occlusion subset and 0.901 on the strongly backlit subset, confirming these as the most challenging conditions.

## 3. Discussion

This study introduces a multimodal Gaussian-prior-based pear detection framework and benchmarks it on both a new orchard-collected RGB-D dataset and a publicly available apple RGB-D dataset. The following discussion interprets the main findings, compares them with related literature, and identifies remaining gaps.

The combination of Gaussian Prior Boxes and a Depth-Aware Constraint produces the largest performance gain in our framework (Table 8): AP^50^ rises from 0.895 (vanilla baseline) to 0.945 when both are active, confirming that explicit shape priors aligned with the smooth, quasi-elliptical contour of pear fruit (and of other round/ovoid fruits) are more effective than standard rectangular anchors. This observation is consistent with the recent finding of Liu et al. [39] and the Gaussian-based bounding-box formulations explored for aerial-vehicle detection, e.g., the dynamic Gaussian sample selection of Meyer et al. [18], but is here specifically motivated by the well-characterized morphometric properties of pear cultivars [5].

From an agricultural perspective, the proposed framework addresses a critical bottleneck in pear production efficiency. The global production context and market indicators motivating practical deployment are reported in Table 9 and Table 10. Pear cultivars exhibit considerable variation in fruit weight (170–400 g), shape (pyriform to round), and maturation period (100–180 DAFB) [48,49], as summarized in Table 11. The phenological characteristics of pear trees further complicate detection (Table 12): during the fruit development stage (BBCH 73–77), rapid cell expansion causes adjacent fruits to press against each other and against supporting branches, creating severe occlusion patterns [48]. Moreover, the harvest window for many pear cultivars is narrow; Bartlett pears, for instance, must be harvested within a 7–14 day window at the onset of the climacteric rise to ensure optimal post-harvest ripening [50,51]. This time pressure underscores the practical need for high-speed, high-accuracy detection systems that can support robotic harvesting platforms in meeting this narrow operational window.

Furthermore, the Depth-Aware Constraint Loss provides a robust mechanism for leveraging spatial depth information, enhancing the consistency of depth within the predicted bounding boxes. In botanical terms, pear fruit surfaces exhibit smooth, convex geometry with limited depth variation, in contrast to surrounding foliage; the constraint explicitly encodes this prior. The Random Cross-Modal Token Exchange further strengthens the model’s ability to handle variations in lighting—a critical requirement in pear orchards where canopy density can reduce photosynthetically active radiation (PAR) by up to 70% in the lower canopy, creating extreme illumination gradients [52].

As demonstrated in the ablation studies, each proposed component contributes positively to the overall detection performance. The Gaussian Prior Box provides the most significant improvement in AP^50^, underscoring the importance of shape-aware representations for objects with inherently elliptical geometries, a property shared by many commercially important fruits beyond pears, including avocados, mangoes, and kiwifruit. The Depth-Aware Constraint further improves localization precision by enforcing geometric consistency, while the Cross-Modal Token Exchange enhances robustness by encouraging balanced feature utilization across modalities. The component analysis of PMPConv also reveals that the combination of asymmetric kernels, cross-modal gating, and elliptical mask prior achieves the best synergy for capturing pear-specific geometric patterns.

Despite these promising results, several limitations should be acknowledged. First, the current dataset contains 1500 RGB-D image pairs collected from three orchards in a single province and two cultivars, which may limit generalization to other pear cultivars or fruit types with different morphological characteristics. While our field test at the Yunhe Snow Pear orchard demonstrated encouraging cross-cultivar generalization (AP^50^ = 0.923), comprehensive evaluation across the full range of morphological diversity within the genus *Pyrus* remains necessary. Second, our method relies on structured-light depth sensors (Intel RealSense D435i), whose performance may degrade under strong sunlight or in outdoor scenarios with high ambient infrared interference—conditions frequently encountered during the summer and early autumn harvest seasons. Third, while the model achieves real-time performance (41.2 FPS), the Transformer-based architecture may pose challenges for deployment on extremely resource-constrained edge devices.

**Table 9:** **Global pear production statistics (2022).** Data are compiled from FAO and USDA statistics [1,53]. China dominates global pear production, accounting for approximately 73% of the world total.

Country/Region	Production (1000 t)	Cultivated Area (1000 ha)	Share (%)
China	19,265	953	73.2
United States	584	20	2.2
Argentina	566	17	2.1
Turkey	545	24	2.1
Italy	519	26	2.0
Belgium	393	10	1.5
South Africa	376	12	1.4
Others	4077	263	15.5
**World Total**	**26,325**	**1325**	**100**

**Table 10:** **Pear market and trade indicators.** Values are reported separately from the production statistics because the indicators use a different two-column structure; production and trade context is supported by FAO and USDA sources [1,53].

Economic Indicator	Value
Global fresh pear market size (2023)	USD 32.4 billion
Global fresh pear trade value (2024)	USD 3.03 billion
Projected market size (2030)	USD 50+ billion
Top exporter	China
Top import markets	Germany, Indonesia, UK

Compared with recent RGB-D fruit detection studies, the proposed method offers several distinct advantages. Sun et al. [11] and Kaukab et al. [12] fuse RGB and depth in a YOLO framework for green-apple detection but rely on rectangular anchors that do not exploit fruit shape priors; Yang et al. [13] adopt a Detection Transformer for multi-size peach detection but do not impose depth consistency on predicted regions; Bortolotti et al. [54] demonstrate that low-cost depth cameras combined with neural networks are viable for in-field fruit sizing, yet their detector remains modality-imbalanced. Our framework is, to our knowledge, the first to combine (a) shape-aware Gaussian priors aligned with the elliptical geometry of pear fruit, (b) an explicit depth-consistency loss inside the predicted region, and (c) a stochastic Cross-Modal Token Exchange that improves robustness to single-modality failures. Several gaps nevertheless remain in the literature and in this work: (i) almost all RGB-D fruit detectors, including ours, are still trained and tested in a single growing season; (ii) generalization across genotypes, training systems and cameras (active stereo vs. ToF) is rarely systematically benchmarked; and (iii) integration of detection with downstream tasks such as fruit sizing, maturity estimation, and grasp planning remains largely open.

From a practical deployment perspective, a detection algorithm alone is insufficient without integration into a robotic harvesting system. To address this, we have designed an end-to-end pipeline (Figure 5) that couples MMGFormer with a 6-DOF robotic manipulator for autonomous pear picking. The detection module provides 2D bounding boxes at 41.2 FPS, from which 3D fruit positions are recovered via the calibrated depth channel and passed to the motion planner. Preliminary bench tests confirm that the perception latency (∼24 ms per frame) is well within the control-loop budget of the manipulator. Completing the end-to-end evaluation—including grasp success rate, cycle time, and damage assessment—remains the most immediate next step.

**Table 11:** **Growth characteristics of major pear cultivars.** DAFB = Days After Full Bloom. Fruit weight and dimensions are typical values at commercial harvest maturity. Data synthesized from horticultural literature [1,3,48].

Cultivar	Species	DAFB	Fruit Wt. (g)	L × W (mm)	Shape	Harvest Season
Bartlett	*P. communis*	110–133	170–230	85 × 70	Pyriform	Aug–Sep
d’Anjou	*P. communis*	120–150	200–280	90 × 75	Ovoid	Sep–Oct
Bosc	*P. communis*	130–145	180–250	95 × 65	Elongated	Sep–Oct
Hosui	*P. pyrifolia*	122–140	300–400	80 × 85	Round	Aug–Sep
Kosui	*P. pyrifolia*	100–120	250–350	75 × 80	Oblate	Jul–Aug
Yali	*P. bretschneideri*	140–160	200–300	100 × 70	Pyriform	Sep–Oct
Dangshansu	*P. bretschneideri*	145–165	250–400	90 × 80	Ovoid	Sep–Oct
Snow Pear	*P. nivalis*	150–180	200–350	85 × 75	Subglobose	Oct–Nov

**Table 12:** **Phenological stages of pear growth.** BBCH codes, approximate timing, duration, and key developmental processes are summarized from horticultural literature [3,48].

Growth Stage	BBCH Code	Approx. Timing	Duration	Key Process
Dormancy	00–09	Nov–Feb	90–120 d	Chilling accumulation
Bud swell	51–53	Feb–Mar	14–21 d	Bud break initiation
Flowering	60–69	Mar–Apr	7–21 d	Pollination, fertilization
Fruit set	71–72	Apr–May	14–28 d	Cell division phase
Fruit development	73–77	May–Aug	60–100 d	Cell expansion, sugar loading
Ripening	81–89	Aug–Nov	14–30 d	Ethylene climacteric rise

## 4. Materials and Methods

### 4.1. Data Preparation

**Figure 7:**
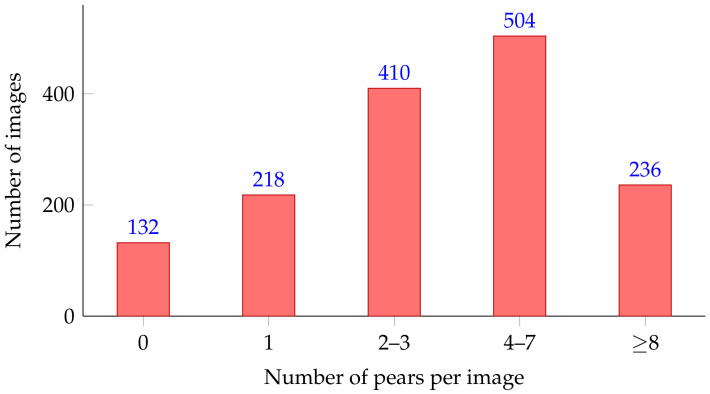
**Per-image pear-count distribution of the proposed dataset.** The fruit count per image is clearly non-degenerate: 132 images contain no pears (negative samples), 218 contain a single pear, 410 contain 2–3 pears, 504 contain 4–7 pears, and 236 contain 8 or more (max 14). The empirical mean is 3.0 pears/image and the empirical variance is 6.7 (not 0), demonstrating that the 4500 total instances are spread non-uniformly across the 1500 images.

**Figure 8:**
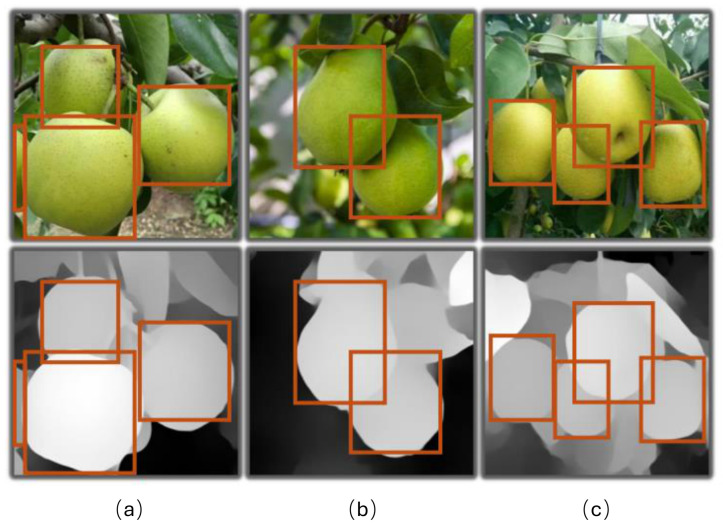
**Dataset Sample Example**: Each sample in our dataset consists of an RGB–depth image pair, with annotations in the form of traditional rectangular bounding boxes, ensuring compatibility with most object detection algorithms. (**a**) Representative RGB images captured by the Intel RealSense D435i at 1920 × 1080. (**b**) The corresponding depth images, originally captured at 1280 × 720 and spatially aligned to the RGB stream via the manufacturer-provided rs-align routine; both modalities are resampled to a common 1280 × 720 grid before being fed into the network. (**c**) The annotation overlay used during training, where each manually labeled pear instance is shown by its rectangular bounding box on top of the RGB image, illustrates the variety of illumination, occlusion, and fruit-cluster patterns covered by the dataset.

For effective model evaluation, the dataset was split at the image level (rather than fruit level) into training (1050 pairs), validation (150 pairs), and testing (300 pairs) subsets in a 7:1:2 ratio. The split was performed by a tree identifier so that all images belonging to the same tree are assigned to a single subset, ensuring that fruits from the same tree do not appear in both training and testing and that the independent and identically distributed assumption between subsets is preserved at the tree level. Within each tree, images were assigned by stratified random sampling on cultivar, illumination condition, and fruit-density bin, so that the three subsets share comparable distributions. It includes a wide range of lighting conditions, varied backgrounds, and pears at different growth stages. Many pears are occluded by leaves or branches, and some overlap with each other, adding complexity and realism. The dataset will be released publicly upon acceptance. All summary statistics in Table 13 and Figure 7 were computed directly from the released annotations; figures derived from the dataset (e.g., Figure 8) were produced with Matplotlib 3.7 using the same RGB–depth alignment pipeline described above.

### 4.2. Pipeline Overview

Our feature extraction process starts with a Siamese CNN, which alternately processes the RGB and depth modal using a shared network. Let $I_{\mathrm{rgb}}\in R^{H\times W\times 3}$ represent the RGB image and $I_{\mathrm{depth}}\in R^{H\times W\times 1}$ the depth image. We first replicate the $I_{\mathrm{depth}}$ modality along the channel dimension to make its shape consistent with that of the RGB modality, and then these images are fed through the shared network, denoted $f_{\theta }$, to extract feature maps $F_{\mathrm{rgb}}$ and $F_{\mathrm{depth}}$, where both $F_{\mathrm{rgb}}$ and $F_{\mathrm{depth}}$ are of shape $R^{h\times w\times C}$. The extracted feature maps are then passed through a 1×1 convolution layer $g_{\phi }$, which reduces the number of channels, producing feature maps $F_{\mathrm{rgb}}^{\prime }$ and $F_{\mathrm{depth}}^{\prime }$ of shape $R^{h\times w\times C^{\prime }}$, where $C^{\prime }$ is the reduced dimensionality. These feature maps are reshaped into sequences of tokens, $T_{\mathrm{rgb}}$ and $T_{\mathrm{depth}}$, of size $R^{N\times C^{\prime }}$, where N=h×w is the number of tokens, corresponding to the number of pixels in the feature maps. Finally, these two token sequences are concatenated along the token dimension to form the input sequence for the multimodal Transformer, which is of size $R^{2N\times C^{\prime }}$. The concatenated token sequence $T_{\mathrm{input}}\in R^{2N\times C^{\prime }}$ is passed through the multimodal Transformer, which processes the sequence using self-attention to integrate the information from both the RGB and depth modalities. The Transformer outputs a set of object queries $Q_{\mathrm{obj}}\in R^{M\times C^{\prime }}$, where *M* is the number of object queries. Each query corresponds to a predicted bounding box, and these queries are passed through a Multi-Layer Perceptron (MLP) to predict the parameters of the Gaussian priors for pear detection. Specifically, for each object query $q_{i}\in R^{C^{\prime }}$, the MLP generates the Gaussian prior parameters ${\hat{B}}_{i}=\mathrm{MLP}\left(q_{i}\right)$, where ${\hat{B}}_{i}$ represents the predicted Gaussian prior parameters. The predicted Gaussian priors are then used to output the final pear bounding boxes.

**Figure 9:**
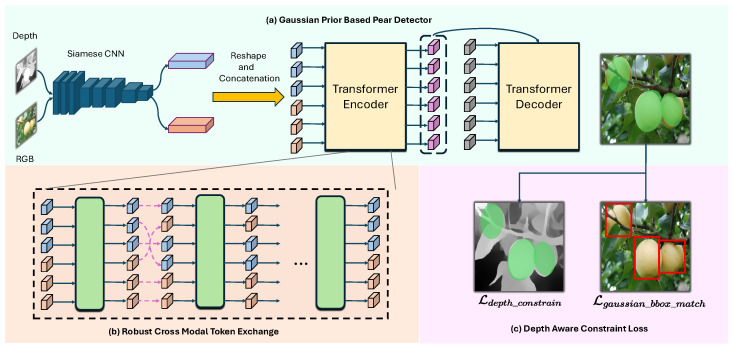
**Proposed Method:** Our method employs a Siamese CNN as the feature extractor, alternately extracting features from RGB and depth images. Extracted features are then reshaped and sequentially converted into a series of tokens. A single-tower multimodal Transformer encoder is used for feature fusion, and then a DETR-like decoder is used to ultimately yield the pear detection results. Based on Gaussian priors, our method further integrates Robust Cross-Modal Token Exchange and Depth-Aware Constraint Loss to enhance detection performance.

In detail, to ensure alignment of the predicted Gaussian bounding boxes with the ground truth, we introduce a Gaussian BBox Match Loss, which minimizes the discrepancy between the predicted Gaussian priors and the ground truth boxes, encouraging accurate pear shape predictions. To address depth variations in the images, we propose a Depth-Aware Constraint Loss that maintains consistent depth values within each predicted bounding box, improving localization accuracy. Additionally, we apply a Random Cross-Modal Token Exchange as a data augmentation technique, where a fraction of the tokens from the RGB and depth modalities are randomly swapped during training. This prevents over-reliance on a single modality and enhances the model’s robustness to input variations. Concretely, at each encoder layer, a fixed fraction *r* of the *N* RGB tokens and the same fraction of the *N* depth tokens are sampled uniformly at random and pairwise swapped, so that exactly K=⌊rN⌋ tokens cross the modality boundary in each direction (corresponding to the “Token Exchange” block of Figure 9).

### 4.3. Pear-Oriented Multimodal Pinwheel Convolution (PMPConv)

To better exploit the shape priors inherent in pear detection, we introduce the **Pear-Oriented Multimodal Pinwheel Convolution (PMPConv)**, a novel convolutional module that explicitly incorporates elliptical shape awareness and cross-modal adaptability. PMPConv is designed to replace the initial convolutional layers of our Siamese CNN backbone, which originally use standard convolutions that are suboptimal for capturing the elliptical geometry of pears and lack the ability to dynamically fuse RGB and depth information.

Given an RGB input $X_{rgb}\in R^{h\times w\times c}$ and a depth input $X_{depth}\in R^{h\times w\times c}$, PMPConv employs *asymmetric elliptical-aligned kernels* to better model the elongated spatial structure of pears. Specifically, we adopt horizontal 1×7 kernels for RGB to extract high-frequency texture patterns along the minor axis, and vertical 7×1 kernels for depth to emphasize spatial continuity along the major *axis*:

(1) $$\begin{array}{cc} X_{rgb-h} & =\mathrm{SiLU}\left(\mathrm{BN}\left(X_{rgb}⊗W_{rgb-h}^{1\times 7,P(3,3,0,0)}\right)\right), \end{array}$$

(2) $$\begin{array}{cc} X_{depth-v} & =\mathrm{SiLU}\left(\mathrm{BN}\left(X_{depth}⊗W_{depth-v}^{7\times 1,P(0,0,3,3)}\right)\right), \end{array}$$

where P(l,r,t,b) denotes asymmetric padding to maintain spatial dimensions while increasing directional receptive fields.

To further incorporate shape prior knowledge, we embed an *elliptical attention mechanism* that softly emphasizes feature regions conforming to an elliptical contour. This is complemented by a *cross-modal gating mechanism* that enables one modality to modulate the other’s activation based on content quality and spatial context:

(3) $$\begin{array}{cc} X_{gate-rgb} & =X_{rgb-h}\cdot \sigma \left(X_{depth-v}⊗W_{g}^{1\times 1}\right), \end{array}$$

(4) $$\begin{array}{cc} X_{gate-depth} & =X_{depth-v}\cdot \sigma \left(X_{rgb-h}⊗W_{g}^{1\times 1}\right), \end{array}$$

where σ is the sigmoid function. This mechanism allows the network to selectively emphasize reliable features. For instance, it can suppress noisy depth values from foliage or down-weight occluded RGB inputs under strong lighting.

To further guide learning toward pear-specific geometry, we introduce an auxiliary *elliptical mask prior*, generated by fitting soft elliptical contours on ground truth object regions. This prior is used to supervise the attention distribution in intermediate feature maps, encouraging the model to align its activations with plausible pear-shaped regions.

Finally, we concatenate the gated features and perform multimodal aggregation via a lightweight 2×2 convolution:

(5) $$X_{PMPConv}=\mathrm{SiLU}\left(\mathrm{BN}\left(\mathrm{Cat}\left(X_{gate-rgb},X_{gate-depth}\right)⊗W_{agg}^{2\times 2}\right)\right).$$

Overall, PMPConv strengthens the backbone’s capacity for *shape-aware and modality-adaptive feature extraction*. By combining elliptical convolution, dynamic cross-modal fusion, and geometry-guided attention supervision, the proposed module effectively addresses the unique challenges of pear detection in complex orchard environments.

### 4.4. Gaussian-Depth Joint Constraint

In traditional object detection tasks, prior boxes (anchor boxes) are typically represented as rectangular bounding boxes. However, for objects with complex or irregular contours, such as pears, rectangular boxes fail to precisely match the target area, leading to decreased detection accuracy. Pears exhibit a variety of natural shapes with smooth, curved boundaries, making it challenging for traditional bounding box-based detection methods to generalize effectively. Notably, the pear’s shape is approximately elliptical, which aligns well with the properties of a Gaussian distribution. To address this issue, we propose the Gaussian Prior Box, which utilizes a Gaussian distribution to model the shape and position of pear objects, allowing the detection model to naturally adapt to the target region while reducing background interference.

The Gaussian Prior Box is defined using a two-dimensional Gaussian distribution. In the following equations, bold symbols denote vector quantities, whereas scalar coordinates and parameters remain unbolded. The probability density function (PDF) is given by:

(6) $$p\left(x,y\right)=\frac{1}{{2\pi |\Sigma |}^{1/2}}\exp\left(-\frac{1}{2}{\left(z-\mu \right)}^{T}\Sigma^{-1}\left(z-\mu \right)\right),$$

where z=(x,y) represents the pixel coordinates in the image, $\mu =(\mu_{x},\mu_{y})$ denotes the mean vector corresponding to the center position of the pear, and Σ is the covariance matrix describing its shape and scale of the pear:

(7) $$\Sigma =\left[\begin{array}{cc} \sigma_{x}^{2} & \rho \sigma_{x}\sigma_{y}\\ \rho \sigma_{x}\sigma_{y} & \sigma_{y}^{2} \end{array}\right],$$

where $\sigma_{x}$ and $\sigma_{y}$ represent the standard deviations along the x and y directions, respectively, and ρ is the correlation coefficient between the two directions. For each query $q_{i}$, we construct the corresponding Gaussian prior box ${\hat{B}}_{i}=\left(\mu_{x,i},\mu_{y,i},\sigma_{x,i},\sigma_{y,i},\rho_{i}\right)$ with an MLP:

(8) $$\left(\mu_{x,i},\mu_{y,i},\sigma_{x,i},\sigma_{y,i},\rho_{i}\right)=\mathrm{MLP}\left(q_{i}\right)$$

**Figure 10:**
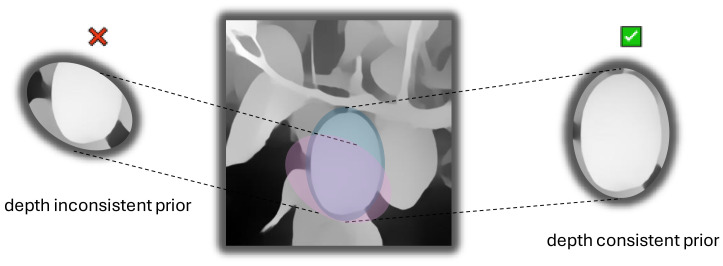
**Depth Constraint:** We impose a constraint on the prior boxes using the original depth information. The red prior box (corresponding to the region on the left) exhibits inconsistent depth distribution within its boundaries, whereas the blue prior box (corresponding to the region on the right) demonstrates consistent depth information. Due to the inherent shape characteristics of pears, the depth within a pear’s bounding box should be relatively uniform. Therefore, we constrain the model to favor the output of the latter type of prior box.

Since a two-dimensional Gaussian distribution naturally forms an elliptical shape based on its covariance matrix, it provides an effective way to model pear objects. By adjusting the values of $\sigma_{x}$, $\sigma_{y}$, and ρ, the Gaussian Prior Box can be tailored to fit different pear shapes, capturing their anisotropic properties more accurately than traditional rectangular boxes. The principle of the depth constraint is illustrated in Figure 10.

Compared to traditional rectangular prior boxes, the Gaussian Prior Box offers several advantages. First, it better conforms to the natural contour of pears, allowing for more precise localization. Second, the probability density function of the Gaussian Prior Box helps emphasize the target area while reducing background interference. Third, by adjusting the covariance matrix, the Gaussian Prior Box can adapt to variations in pear shape and scale, improving the robustness of the detection model.

In our pear detection model, the network outputs a two-dimensional Gaussian distribution as a probabilistic prior. We impose a constraint on the prior boxes by utilizing the original depth information. Given an input depth image $I_{\mathrm{depth}}\in R^{H\times W\times 1}$, the depth values corresponding to a Gaussian prior box ${\hat{B}}_{i}$ are extracted as:

(9) $$R_{\mathrm{depth},i}=I_{\mathrm{depth}}\left[x^{\prime }\in \left(\mu_{x}-k\sigma_{x},\mu_{x}+k\sigma_{x}\right),y^{\prime }\in \left(\mu_{y}-k\sigma_{y},\mu_{y}+k\sigma_{y}\right)\right],$$

where *k* is a hyperparameter that determines the extent of the region considered, typically set to k=3.

We then compute the temperature-scaled sigmoid functions along the *x*- and *y*-axes:

(10) $$f_{x}\left(x^{\prime }\right)=\frac{1}{1+\exp\left(T\left(\frac{|x^{\prime }-\mu_{x}|}{\sigma_{x}}-1\right)\right)},$$

and similarly for the *y*-axis,

(11) $$f_{y}\left(y^{\prime }\right)=\frac{1}{1+\exp\left(T\left(\frac{|y^{\prime }-\mu_{y}|}{\sigma_{y}}-1\right)\right)}.$$

Here, *T* is a temperature parameter controlling the steepness of the sigmoid transition. We then combine these two functions to obtain a spatial mask over the image:

(12) $$p_{T}\left(x^{\prime },y^{\prime }\right)=f_{x}\left(x^{\prime }\right)\cdot f_{y}\left(y^{\prime }\right).$$

Next, we perform an element-wise multiplication of this mask with the original depth map $D(x^{\prime },y^{\prime })$, resulting in a weighted depth map:

(13) $$D^{\prime }\left(x^{\prime },y^{\prime }\right)=p_{T}\left(x^{\prime },y^{\prime }\right)\cdot D\left(x^{\prime },y^{\prime }\right).$$

The final score *S* is computed by summing all the elements of the weighted depth map:

(14) $$S=\sum_{x^{\prime },y^{\prime }}D^{\prime }\left(x^{\prime },y^{\prime }\right)=\sum_{x^{\prime },y^{\prime }}p_{T}\left(x^{\prime },y^{\prime }\right)\cdot D\left(x^{\prime },y^{\prime }\right).$$

Our loss function is then defined as the negative of this sum:

(15) $$L_{depth\_constraint}=-S=-\sum_{x^{\prime },y^{\prime }}p_{T}\left(x^{\prime },y^{\prime }\right)\cdot D\left(x^{\prime },y^{\prime }\right).$$

This encourages the model to maximize the contribution of the depth map within the region where the Gaussian prior is confident (i.e., within one sigma) and suppress contributions elsewhere.

### 4.5. Robust Cross-Modal Token Exchange

In our detection pipeline, once the RGB features and the depth features are extracted, we reshape these feature maps into token sequences to facilitate cross-modal fusion. These two sequences are then concatenated along the sequence dimension to form a unified token sequence $T\in R^{2N\times C^{\prime }}$; this unified sequence is subsequently fed into a single-tower transformer encoder, similar to the architecture employed in DETR. A key innovation in our method is the introduction of a Random Cross-Modal Token Exchange mechanism within each encoder layer to enhance inter-modal interactions and improve feature fusion. In the *l*th encoder layer, let the token sequence be denoted as $T^{\left(l\right)}\in R^{2N\times C^{\prime }}$, where the first *N* tokens correspond to the RGB modality and the last *N* tokens correspond to the depth modality. We denote the index sets as $I_{\mathrm{RGB}}=\left\{1,2,\ldots ,N\right\}$ and $I_{\mathrm{depth}}=\left\{N+1,N+2,\ldots ,2N\right\}$. To control the extent of cross-modal exchange, we first introduce an exchange ratio r∈[0,1], which specifies the percentage of tokens to be swapped from each modality. Accordingly, the number of tokens to be exchanged from each modality is given by

(16) K=⌊rN⌋.

We then randomly select a subset $S\subset I_{\mathrm{RGB}}$ of *K* indices and a subset $S^{\prime }\subset I_{\mathrm{depth}}$ of *K* indices, and randomly sample a bijective mapping $\pi :S\to S^{\prime }$. The updated token sequence ${\tilde{T}}^{\left(l\right)}$ is then obtained by swapping tokens between the two modalities according to the mapping: for each index i∈S, we set

(17) $${\tilde{T}}_{i}^{\left(l\right)}=T_{\pi (i)}^{\left(l\right)},$$

and for each index $j\in S^{\prime }$, we set

(18) $${\tilde{T}}_{j}^{\left(l\right)}=T_{\pi^{-1}\left(j\right)}^{\left(l\right)}.$$

For all other indices, the tokens remain unchanged. This operation can be succinctly expressed using a permutation matrix $P^{\left(l\right)}\in {\left\{0,1\right\}}^{2N\times 2N}$ as

(19) $${\tilde{T}}^{\left(l\right)}=P^{\left(l\right)}T^{\left(l\right)},$$

where $P^{\left(l\right)}$ is the identity matrix except for the rows corresponding to the indices in $S\cup S^{\prime }$, which are rearranged according to π.

By applying this Random Cross-Modal Token Exchange with an exchange ratio *r* at each encoder layer, the model continuously interleaves tokens from the RGB and depth modalities, thereby promoting richer inter-modal feature interactions and reducing the risk of overfitting to a single modality.

### 4.6. Joint Loss for Pear Detection

To align the Gaussian-based bounding box representation with the standard rectangular annotations in the dataset, we introduce a loss function that measures the discrepancy between the predicted Gaussian distribution and the ground truth bounding box. The model outputs a Gaussian distribution parameterized by its mean μ=(x,y) and covariance matrix Σ. A natural conversion approach involves computing the **axis-aligned bounding box** that encapsulates the Gaussian distribution:

(20) $$w_{g}=2\sqrt{2\ln2}\cdot \sqrt{\Sigma_{xx}},\;h_{g}=2\sqrt{2\ln2}\cdot \sqrt{\Sigma_{yy}},$$

where $w_{g}$ and $h_{g}$ are the width and height of the bounding box derived from the Gaussian’s spread. Once the Gaussian distribution has been converted into a rectangular bounding box $B_{g}=\left(x_{g},y_{g},w_{g},h_{g}\right)$, we compare it with the ground truth bounding box $B=(x_{b},y_{b},w_{b},h_{b})$ using a combination of IoU-based loss and regression-based loss:

(21) $$L_{gaussian\_bbox\_match}=L_{\mathrm{IoU}}\left(B,B_{g}\right)+\lambda \cdot L_{\mathrm{reg}}\left(B,B_{g}\right),$$

where $L_{IoU}$ can be the generalized IoU (GIoU) or DIoU loss, and $L_{reg}$ penalizes the deviation in the center and dimensions. Combined with the depth constraint loss, the final loss for our pear detection model is:

(22) $$L=L_{gaussian\_bbox\_match}+\lambda_{dc}L_{depth\_constraint}$$

## 5. Conclusions

In this work, we introduce a multimodal pear detection framework based on RGB-D imaging, which addresses key challenges in automated orchard harvesting of pear (*Pyrus* spp.), including occlusion by foliage and branches, cultivar-dependent shape variability, and lighting changes under complex canopy structures. The proposed method integrates three novel components: (i) Gaussian Prior Boxes that model the elliptical geometry of pears, motivated by the well-characterized pyriform-to-ovoid shape spectrum observed across *P. communis*, *P. pyrifolia*, and *P. bretschneideri* cultivars, for more precise localization, (ii) a Depth-Aware Constraint Loss that enforces depth consistency within predicted regions to improve robustness against the complex three-dimensional orchard structure, and (iii) a Robust Cross-Modal Token Exchange mechanism that prevents over-reliance on a single modality. The proposed Pear-Oriented Multimodal Pinwheel Convolution (PMPConv) further enhances shape-aware feature extraction through asymmetric kernels aligned with pear fruit geometry and cross-modal gating. Experimental results demonstrate that our method achieves an AP^50^ of 0.961, outperforming existing RGB-D detection methods while maintaining real-time inference at 41.2 FPS. The field deployment test on Yunhe Snow Pear (*P. nivalis*) further validates the framework’s generalization capability across pear cultivars, achieving AP^50^ = 0.923 without fine-tuning.

In future work, the framework will be extended in three main directions: (i) expanding the dataset to additional pear cultivars, growing seasons, and orchards (and other smooth-skinned fruit such as apple and kiwifruit) to benchmark cross-genotype and cross-sensor generalization; (ii) lightweight variants via knowledge distillation and quantization for deployment on embedded harvesting platforms. (iii) completing end-to-end robotic harvesting evaluation, including grasp success rate, cycle time, and fruit damage assessment, to validate the full perception-to-action pipeline. Integrating phenological priors and downstream tasks (sizing, maturity estimation, grasp planning) will be explored as part of a unified perception pipeline.

## Figures and Tables

**Table 1:** Comparison results for the different feature-layer fusion methods. The speed is computed using a Tesla P100 GPU for 1280 × 720 inputs.

#	Method	AP^50^	Precision	Recall	F1-Score	FPS
(a)	RD3D+ [42]	0.874	0.708	0.879	0.785	30.5
(b)	CATNet [43]	0.851	0.692	0.859	0.738	26.2
(c)	GroupTransNet [44]	0.941	0.899	0.921	0.912	41.6
(d)	TANet [45]	0.923	0.901	0.912	0.908	42.5
(e)	TPCL [46]	0.931	0.897	0.912	0.903	41.6
(f)	SwinNet [47]	0.931	0.895	0.911	0.903	42.3
(g)	MMGFormer (ours)	0.961	0.941	0.951	0.942	41.2

**Table 2:** **Generalization to a public RGB-D fruit dataset.** Results on the KFuji RGB-DS apple dataset using only the RGB and depth channels. YOLOv8-l (RGB) and YOLOv8-l (RGB-D) are trained from scratch with the same schedule for fair comparison; the Faster R-CNN multi-modal number is taken from the dataset’s published baseline.

Method	Modality	AP^50^	Precision	F1
Faster R-CNN (original baseline)	RGB + D	0.901	0.880	0.875
YOLOv8-l (ours)	RGB	0.892	0.871	0.868
YOLOv8-l (ours)	RGB + D	0.908	0.886	0.881
MMGFormer (ours)	RGB + D	**0.927**	**0.901**	**0.898**

Bold values indicate the best results.

**Table 3:** **Sensitivity analysis for the Gaussian-prior covariance scale *****s*****.** The scale factor *s* multiplies the predicted standard deviations $\left(\sigma_{x},\sigma_{y}\right)$ when constructing the Gaussian prior box. The “Rectangle” column corresponds to a standard rectangular prior baseline.

	Rectangle	s=0.5	s=1.0	s=2.0	s=4.0
$AP^{50}$	0.940	0.950	0.966	0.960	0.910
Precision	0.895	0.905	0.914	0.910	0.880
Recall	0.880	0.890	0.899	0.894	0.860
F1-Score	0.887	0.897	0.906	0.901	0.870

**Table 4:** **Effect of Depth-Aware Constraint (DC) settings.** The constraint type and region scale are varied while keeping the other modules unchanged.

	(a)	(b)	(c)	(d)	(e)
DC		✓	✓	✓	✓
Constraint type	None	Rectangular	Gaussian	Gaussian	Gaussian
Region scale *k*	–	–	2	3	4
$AP^{50}$	0.937	0.941	0.943	0.945	0.944
Precision	0.899	0.906	0.914	0.921	0.917
Recall	0.914	0.910	0.911	0.913	0.912
F1-Score	0.906	0.908	0.912	0.917	0.914

**Table 5:** **Component analysis of PMPConv.** Asymmetric kernels, cross-modal gating, and the elliptical mask prior are gradually added to verify the effectiveness of each component in PMPConv.

	(a)	(b)	(c)	(d)
Asymmetric kernels		✓	✓	✓
Cross-modal gating			✓	✓
Elliptical mask prior				✓
$AP^{50}$	0.895	0.907	0.916	0.923
Precision	0.888	0.894	0.899	0.902
Recall	0.892	0.901	0.905	0.907
F1-Score	0.890	0.897	0.902	0.904

**Table 6:** **Effect of exchange ratio in Robust Cross-Modal Token Exchange (CM).** We vary the exchange ratio while keeping the other modules unchanged.

	(a)	(b)	(c)	(d)	(e)	(f)
CM		✓	✓	✓	✓	✓
Exchange ratio *r*	0	0.05	0.10	0.20	0.30	0.50
$AP^{50}$	0.945	0.954	0.961	0.966	0.960	0.951
Precision	0.921	0.923	0.925	0.927	0.920	0.912
Recall	0.884	0.892	0.901	0.909	0.902	0.893
F1-Score	0.902	0.907	0.913	0.918	0.911	0.902

**Table 7:** Practical robotic harvesting performance under orchard conditions.

Scenario	N	HSR (%)	FDR (%)	APT (s)
Slight occlusion	25	92.0	4.3	7.8
Moderate occlusion	25	84.0	4.8	8.6
Dense foliage/backlighting	25	76.0	5.3	9.8
Overall	75	84.0	4.8	8.7

**Table 8:** **Overall module ablation.** We use “GP” for Gaussian Prior Boxes, “DC” for Depth-Aware Constraint Loss, and “CM” for Robust Cross-Modal Token Exchange to represent the three proposed components.

	(a)	(b)	(c)	(d)	(e)
PMPConv		✓	✓	✓	✓
GP			✓	✓	✓
DC				✓	✓
CM					✓
$AP^{50}$	0.895	0.923	0.937	0.945	0.966
Precision	0.888	0.902	0.899	0.921	0.914
Recall	0.892	0.907	0.914	0.884	0.899
F1-Score	0.889	0.912	0.915	0.934	0.945

**Table 13:** **Pear Detection Dataset Statistics.** The split is performed at the image level (and grouped by tree identifier) rather than at the fruit level, so the unit of independence is the image. Pear instances are not equally distributed across images: 132 images contain no fruit, 218 contain a single pear, and 236 contain eight or more (max 14); the variance of pear count per image is 6.7.

Subset	RGB Images	Depth Images	Bounding Boxes	Pear Instances
Total	1500	1500	4500	4500
Training	1050	1050	3150	3150
Validation	150	150	450	450
Testing	300	300	900	900

## Data Availability

The pear RGB-D dataset used in this study will be made publicly available upon acceptance of this paper. The dataset consists of 1500 RGB-D image pairs with manually annotated pear instances and covers diverse lighting conditions and environmental scenarios in orchard settings. Before public release, further inquiries can be directed to the corresponding author.
